# Challenges and applications of artificial intelligence in infectious diseases and antimicrobial resistance

**DOI:** 10.1038/s44259-024-00068-x

**Published:** 2025-01-07

**Authors:** Angela Cesaro, Samuel C. Hoffman, Payel Das, Cesar de la Fuente-Nunez

**Affiliations:** 1 Machine Biology Group, Department of Psychiatry and Microbiology, Institute for Biomedical Informatics, Institute for Translational Medicine and Therapeutics, Perelman School of Medicine, University of Pennsylvania, Philadelphia, PA USA; 2 Department of Bioengineering and Chemical and Biomolecular Engineering, School of Engineering and Applied Science, University of Pennsylvania, Philadelphia, PA USA; 3 Department of Chemistry, School of Arts and Sciences, University of Pennsylvania, Philadelphia, PA USA; 4 Penn Institute for Computational Science, University of Pennsylvania, Philadelphia, PA USA; 5 IBM Research, Thomas J. Watson Research Center, Yorktown Heights, New York, NY USA

**Keywords:** Drug discovery, Machine learning

## Abstract

Artificial intelligence (AI) has transformed infectious disease control, enhancing rapid diagnosis and antibiotic discovery. While conventional tests delay diagnosis, AI-driven methods like machine learning and deep learning assist in pathogen detection, resistance prediction, and drug discovery. These tools improve antibiotic stewardship and identify effective compounds such as antimicrobial peptides and small molecules. This review explores AI applications in diagnostics, therapy, and drug discovery, emphasizing both strengths and areas needing improvement.

## Introduction

In recent years, artificial intelligence (AI) has dramatically accelerated the discovery of new antibiotics^1–5^. Whereas with traditional approaches it takes years to discover new antibiotics, with computers this can now be done in hours.

**Fig. 1:**
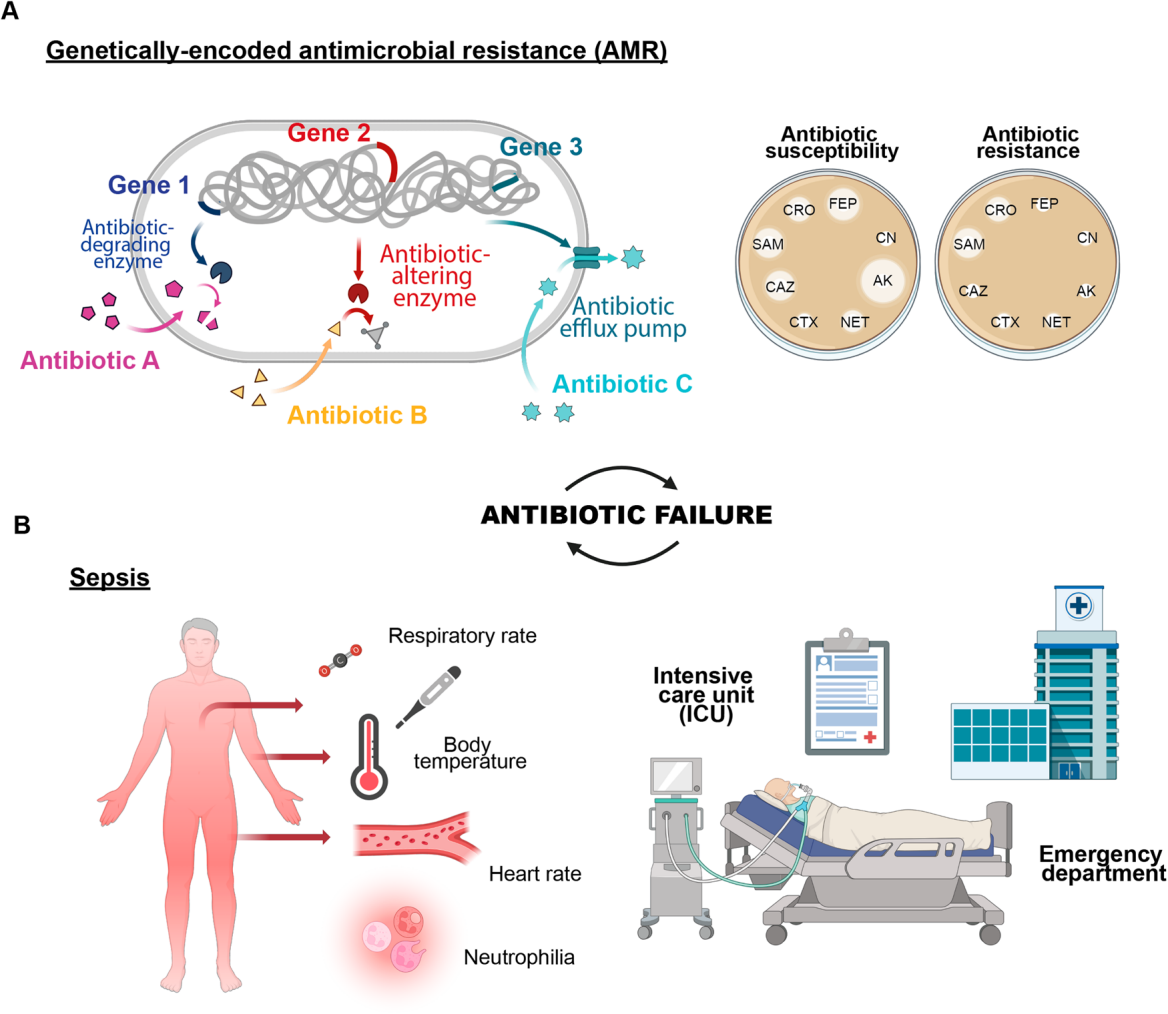
Factors contributing to antibiotic failure: **A** Antimicrobial resistance driven by genetic exchange or mutation, leading to evolving resistance, and (**B**) sepsis, a severe response to infection, intensifying the urgency for effective treatment.

Despite these scientific and technological advances, millions of people continue to die from infections annually. The overuse and misuse of antibiotics has led to the emergence of pan-drug-resistant microbes, resulting in over 4 million deaths in 2019 alone^6,7^. However, the failure of antimicrobial treatments to effectively cure patients and eradicate pathogens is not solely due to antibiotic-resistant genes. Multiple factors, including complex mechanisms underlying critical clinical conditions such as sepsis, contribute to antibiotic failure (Fig. 1). This multifaceted issue has become a growing global concern^6,8,9^.

Recently, the substantial proliferation of medical and biological data has spurred the development of numerous AI-based techniques. These techniques empower computers to learn and adapt to increasingly intricate information^10–14^. In the context of antibiotic failure, AI offers tremendous opportunities for disease diagnosis and drug discovery, particularly in scenarios where traditional antibiotics are ineffective^15–22^.

Machine learning (ML), a branch of AI, has made significant progress with diverse applications in computational biology, language processing, gaming, and computer vision. Some ML models leverage deep learning (DL), an approach employing deep neural networks (DNNs) to extract hidden features and predict outcomes from complex datasets, including images and biomolecular structures^5,10,23–26^.

Cutting-edge algorithms have demonstrated the ability to analyze extensive clinical and experimental data, providing insights into disease diagnosis, treatment prognosis, and the prediction or generation of novel and effective antimicrobial compounds. These computer-aided technologies hold the potential to address clinical conditions caused by the inefficacy of antibiotics, where timely and accurate interventions are critical.

In this review, we explore the use of computer-guided methodologies in diagnostics, therapeutic approaches, and drug discovery, providing examples at various levels. We highlight the technical features and advantages of these methodologies, demonstrating how they enhance efficiency in patient care and treatment development. Additionally, we point out the limitations and challenges associated with these technologies including data availability and ethical concerns, emphasizing the need for standardized datasets and intervention from authorities to ensure data protection.

## Antibiotic failure: an underappreciated global health problem

One of the primary causes of antibiotic failure is the natural selection of antibiotic-resistant phenotypes. A recent report published in January 2022 revealed that six leading bacterial pathogens, including *Escherichia coli*, *Staphylococcus aureus*, *Klebsiella pneumoniae*, *Streptococcus pneumoniae*, *Acinetobacter baumannii*, and *Pseudomonas aeruginosa*, were responsible for millions of global deaths attributable to or associated with antimicrobial resistance (AMR)^7^. The Centers for Diseases Control and Prevention (CDC) highlights a significant and alarming increase in resistant infections occurring during hospitalizations, placing the AMR as one of the major threats to human health today^27^. In 2021, there were 1.5 million deaths directly attributable to AMR and 4.71 million associated death^6^. According to a recent scenario presented by *The Lancet*, the burden of AMR is projected to rise to 1.91 million attributable deaths and 8.22 million associated deaths by 2050. This indicates that, without further interventions, achieving the proposed 10% reduction in AMR mortality by 2030 will be unlikely (Fig. 1A)^6^.

In addition to the challenge of resistance evolution, the process of bringing a new drug to market spans an average of 15 years. This duration encompasses stages such as drug discovery and development, preclinical and clinical trials, regulatory review by the U.S. Food and Drug Administration (FDA), and post-market safety monitoring^28^. Other concerns include estimated costs required to bring a new compound to the market range from $161 million to $4.5 billion^29^. Indeed, traditional drug discovery methods often involve costly and time-consuming high-throughput screenings, which assess numerous potential compound combinations across various bacterial strains. Consequently, the persistent emergence of antibiotic-resistant strains, coupled with the scarcity of new effective antibiotics against resistant species, is placing considerable pressure on healthcare systems. This has sparked interest in integrating state-of-the-art AI models and other computational methodologies to expedite the identification of novel drugs with desired characteristics while enabling the exploration of a wider chemical space.

Genetically-determined antimicrobial resistance, which lead to the selection of strains such as methicillin-resistant *S. aureus* (MRSA), vancomycin-resistant *Enterococcus* (VRE), carbapenem-resistant *Acinetobacter* and extended-spectrum beta-lactamase (ESBL)-producing *Enterobacterales*, is only one of the factors contributing to antibiotic failure. Antibiotic failure is defined as any event where an antimicrobial treatment is no longer effective in eradicating the bacterial infection, leading to the persistence or worsening of the clinical condition^9^. While cases of AMR and mechanisms underlying bacteriostatic activity and drug inactivation are closely related to the performance of the antibiotic, other factors such as pathogen colonization (e.g., biofilm formation) and the body’s response (e.g., sepsis) are influenced by the type of infection and the clinical condition of the patient. These factors contribute equally to antibiotic failure (Fig. 1B)^8,9^.

Bacterial biofilms are colonies of microbes encased in a polymeric matrix, commonly found on surfaces and implicated in about 65% of infections, including those related to medical devices like catheters and implants. Biofilm cells exhibit resistance to antibiotics that is 10 to 1,000 times higher than their planktonic counterparts, due to factors such as restricted antibiotic diffusion and altered gene expression^30^. Additionally, biofilms can evade host defenses, allowing them to persist and contribute to chronic infections^30^. In this context, a prompt detection of bacterial species capable of forming biofilms is crucial for effective treatment and management^8^.

Sepsis is a critical condition defined by an uncontrolled immune response to an infection, with the most severe instances involving dysfunction of multiple organs. Annually, about 1.7 million people develop sepsis in the U.S. alone, with approximately 350,000 dying during hospitalization^31^. Even though these numbers are not comparable to the predicted deaths caused by AMR, individuals who die from sepsis represent 19.7% of all deaths reported annually worldwide^9^. For instance, antibiotics are the primary approach in the management of sepsis, however their effectiveness is limited by high mortality rates (23-35%) and clinical complications. Prolonged or inappropriate use of antibiotics can lead to resistant infections and adverse side effects^32^. The initial symptoms of sepsis are often nonspecific, delaying the identification of the causative pathogen and the initiation of adequate treatment^9^. As a result, initial therapies are often empirical and broad-spectrum, leading to excessive exposure to nonspecific antimicrobial therapies, that is the reason why they are ineffective in more than 20% of cases (Fig. 1B)^32^.

**Fig. 2:**
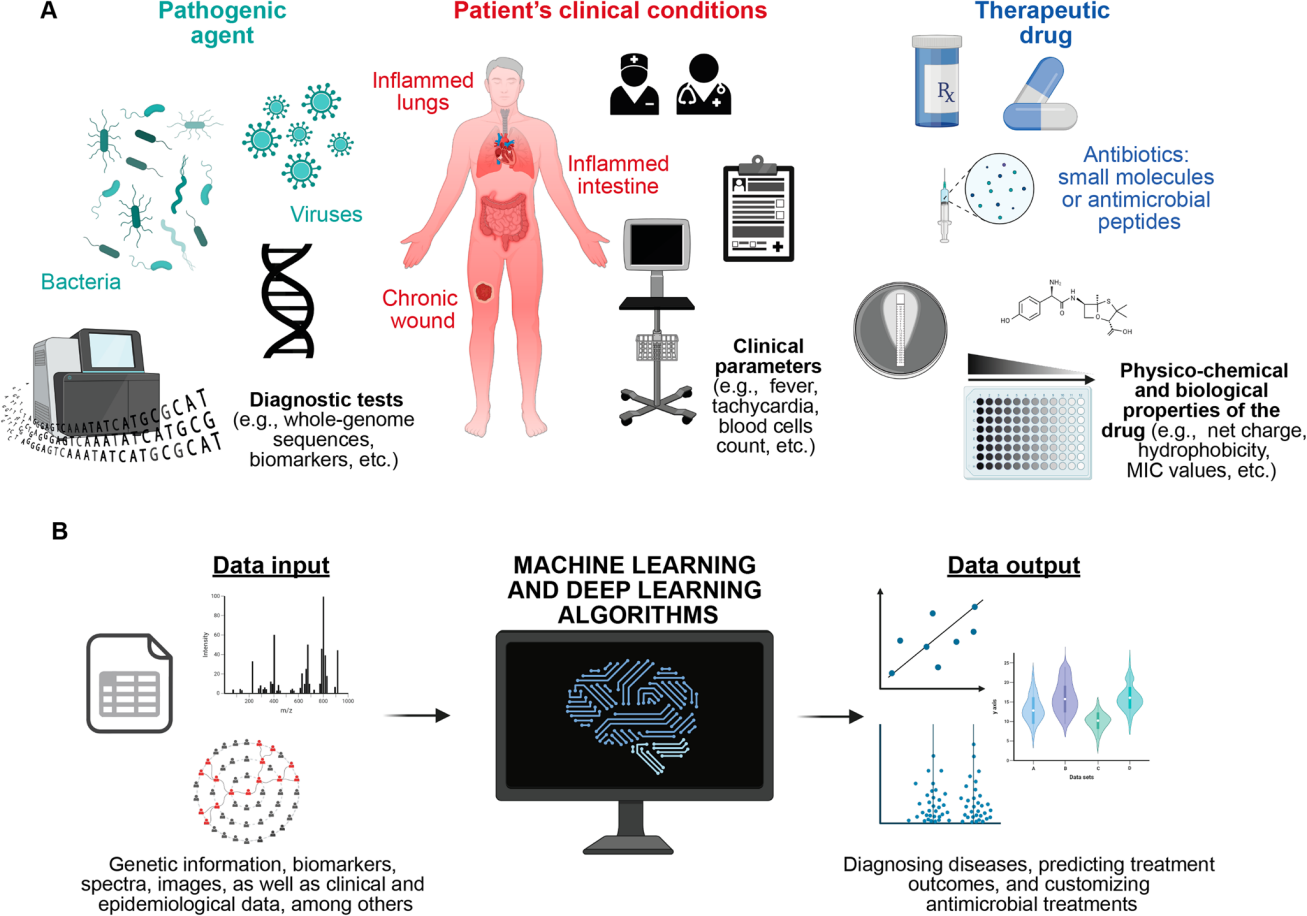
Using AI to combat antibiotic failure. **A** Gathering information and data from diagnostic tests, ICU patient clinical conditions, and antimicrobial effectiveness. **B** Developing AI-powered models for infection diagnosis, predicting antimicrobial resistance, and tailoring personalized antimicrobial therapies.

In this context, AI-based methods have emerged as valuable tools for analyzing extensive datasets and revealing intricate patterns that might elude human observation (Fig. 2A). This facilitates the development of advanced diagnostic tools and personalized treatment plans (Fig. 2B)^33^.

## AI assistance in diagnostics

Although AI is still in its nascent stages for medical diagnosis, increasing data availability is paving the way for its broader application, especially in detecting diseases such as cancer^33^. In the specific case of infectious diseases and sepsis, the complexity of the underlying mechanisms and the symptoms presents a challenge in developing early diagnostic tools. However, ML models hold promise in diagnosis through workflow management, task automation, and decision-making assistance. Additionally, deep learning frameworks, employing convolutional neural networks (CNN), recurrent neural networks (RNN) and data mining, are increasingly playing an important role in identifying disease patterns within extensive datasets^34^. This capability holds immense potential for diagnosing, predicting, and classifying diseases within healthcare systems. A comparison of selected AI models and their potential applications in diagnostics is summarized in Table 1.

**Table 1:** A comparison of selected AI models with potential use in diagnostics

Task	Data Modality	Data source	Model types
Bloodstream infection detection	Time-series	ICU clinical parameters	RNN, LSTM
Gram stain interpretation	Image	Microscopy	CNN
AMR prediction	Image	Microscopy	CNN
	Mass spectra	Mass spectrometry	MLP, GBM
	k-mers	Genome sequencing	LR, RF, SVM
MIC prediction	k-mers	Genome sequencing	GBM

Model types are generally determined by the data modality available, and more possibilities exist than those listed here.

In the critical setting of intensive care units (ICUs), diverse data formats, such as images, numbers, and text, demand a swift and accurate assessment due to patient urgency. Moreover, deciphering intricate and nonlinear data relationships is essential. While traditional statistical tools like linear regression offer insights through mathematical equations by recommending a “best-fit line”, DL methods are able to meticulously analyze evidence without simplifying the complex relationships. DL can simultaneously process multiple inputs, enabling the creation of predictive models tailored to specific outcomes.

For example, Steenkiste et al. applied a bidirectional long short-term memory (LSTM) model to predict blood culture outcomes in ICU patients^35^. LSTMs are a type of RNN meaning the inputs to the model are both the current features as well as the previous state, making it ideal for time-series data. Specifically, LSTMs contain a module to decide what to remember and forget from past states, improving their ability to keep track of long-term dependencies. Their model used nine clinical characteristics, including temperature, blood thrombocyte count, blood leukocyte count, C-reactive protein concentration, sepsis-related organ failure assessment, heart rate, respiratory rate, international normalized ratio of prothrombine time and mean systemic arterial pressure, evaluated over time using a high-quality database of 2,177 ICU patients. The network achieved an area under the receiver operating characteristic (ROC) curve (indicating diagnostic performance) of 0.99 and an average area under the precision-recall curve (AUC) of 0.82, reflecting its accuracy. As a note, the ROC curve provides a visual representation that shows how well a classification model performs across various threshold settings by plotting the true positive rate (sensitivity) against the false positive rate (1-specificity) for different threshold values; the AUC, on the other hand, averages the model’s performance in distinguishing between positive and negative cases, offering a comprehensive view of the model’s overall effectiveness. In addition to the significant ROC and AUC values, Steenkiste et al.’s findings also indicate that predicting several hours before the event results in only a slight decrease in predictive power^35^.

Further application of AI in microbiology can involve processing data such as images, mass spectra, and bacterial whole-genome sequences^16,36^.

Smith et al. employed a pre-trained CNN, initially designed for image classification, and adapted it to identify various bacteria, including different Gram stain morphologies (for example, Gram-positive *cocci* arranged in clusters, Gram-positive *cocci* arranged in chains, and Gram-negative rods) from blood culture samples tested positive. CNNs use sets of learned filters applied across the image and at various scales to provide a rich featurization used for classification or other tasks. A total of approximately 100,000 classified image sections (cropped portions of a Gram-stain slide) were inputted into the CNN^37^. The network attained approximately 95% accuracy in classifying image crops and 92.5% accuracy in classifying entire slides across all categories^37^.

Another example is the recent work of Zagajewski et al., who used CNNs to differentiate the susceptible phenotype from the resistant (untreated) phenotype at the single-cell level, facilitating swift and detailed phenotyping of individual cells that exhibit varied physiological responses to antibiotics^38^.

Conversely, most AI applications utilizing mass spectra focus on comparing the proteomic fingerprint, expressed as the mass-to-charge (m/z) peak values and intensities, of an unknown sample against a curated database of known spectra. Traditional software uses predefined rules to evaluate the similarity between the unknown spectrum and the database entries. In contrast, machine learning methods harness intrinsic data patterns to develop predictive models. This approach is especially effective for predicting antimicrobial resistance and performing strain typing or outbreak investigations, eliminating the need for explicit programming^16,39,40^. For instance, Weis et al. gathered mass spectrometry data from clinical strains and pairing it with resistance profiles, establishing the extensive DRIAMS dataset^10,41^. This dataset includes resistance information for over 70 antimicrobials and mass spectrometry data for more than 300,000 clinical strains across 803 pathogens. Using this dataset, three machine learning algorithms were trained: logistic regression, a deep neural network classifier (multilayer perceptron, MLP), and gradient-boosted decision trees (LightGBM). Among these, the MLP and LightGBM emerged as the most effective classifiers. This innovative method showcases the potential to significantly improve antibiotic stewardship and optimize infection treatment strategies in an efficient and reliable manner^41^.

AI and ML methods also leverage bacterial DNA sequence in conjunction with its antimicrobial susceptibility phenotype^42^. Indeed, ML analyzes a large library of these bacteria isolates, studying the connections between their DNA sequences and antimicrobial resistance profile. Various ML algorithms, such as those analyzing short sequences of DNA (k-mers) and weighting them based on their predictive value for antimicrobial resistance phenotype, are employed^42^. Once these models are developed, they can reliably forecast susceptibility in new isolates during sequencing.

Furthermore, encouraging findings have emerged regarding ML models’ ability to predict not only the phenotypic profile but also the observed minimum inhibitory concentration (MIC) of the antimicrobials under evaluation. For example, Nguyen et al. used an extreme gradient boosting (XGBoost)-based ML model, successfully predicting resistance as well as MIC in nontyphoidal *Salmonella*^43^. Moreover, combining genomes and transcriptomes could also enhance the predictive abilities of ML-based classifiers in foreseeing antimicrobial resistance phenotypes, particularly in Gram-negative ESKAPE pathogens^42^.

In addition to high accuracy, model interpretability is crucial for gaining the trust and acceptance of both patients and clinicians in AI. Explainability methods fall into two main categories: intrinsically interpretable models (or “white-box” models) and post-hoc explanations of “black-box” models. In clinical settings, directly interpretable models are generally preferred since they are easily understood by all stakeholders^44,45^. However, for more complex data, post-hoc methods may be necessary. For instance, Drouin et al. developed a sparse, rule-based model called the Set Covering Machine (SCM) to predict antimicrobial resistance using genome k-mers^46^. On the other hand, Martínez-Agüero et al. combined various recurrent neural networks (RNNs) with post-hoc Shapley additive explanations (SHAP) for early prediction of antimicrobial resistance using ICU electronic health records^47^. SHAP, a model-agnostic tool for local explanations, ranks features by their relevance to an individual prediction and can be applied to many types of models^48^. Despite its utility, there are still many open questions about post-hoc AI explainability methods, as they are inherently imperfect representations of the underlying models. The trade-offs in their use must be carefully considered on a case-by-case basis^49^.

In the future, it is expected that the number of AI tools, the precision and reliability of AI software analyses, the integration of AI into clinical microbiology laboratory protocols, along with advances in model interpretability, will all continue to expand.

## AI for infectious disease treatment

The need for a rapid and accessible method to predict bacterial antimicrobial susceptibilities for informed prescription is substantial in clinical settings, and once more, AI can be helpful in advancing personalized treatment by analyzing data (e.g., genetics, lifestyle, and biomarkers), predicting outcomes with targeted interventions and optimizing clinical strategies^50^.

For instance, Kanjilal et al. developed an ML model using electronic health records of 10,053 patients with uncomplicated urinary tract infections (UTIs) recorded in Boston from 2007 to 2013, suggesting the most precise antibiotic^51^. Tested retrospectively on 3629 patients with uncomplicated UTI presented to the same hospitals between 2014 and 2016, the algorithm outperformed clinicians, slashing second-line antibiotic usage by 67%^51^.

Another example has been demonstrated by Yelin et al. who developed a ML algorithm able to personalize antibiotic prescriptions based on patient demographics, urine culture history, and drug purchase records^52^. Using longitudinal data spanning a decade, they examined a dataset comprising 711,099 instances of non-hospital-acquired UTIs involving 315,047 individual patients. The developed algorithm effectively predicts antimicrobial resistance tailored to specific drugs on a personalized basis, consequently leading to a reduction in the rate of mismatched treatments from over 8% to below 6%^52^.

Another application of AI in health care settings is represented by chatbots, which connect patients to information through natural, human-like language interactions. In the context of infectious diseases, chatbots can play a crucial role in providing timely information about symptoms, prevention, and treatment options^53^. Chatbots typically consist of four core modules: a text comprehension module, a dialog manager, a text generation component, and a database layer for training and operational use^13^. The text understanding module interprets user input using methods like pattern matching [keyword/string matching and Extensible Markup Language (XML) variants like Artificial Intelligence Markup Language (AIML)], machine learning (decision trees and random forests), and natural language processing (NLP) techniques (like named entity recognition)^13^. Some chatbots also use rule-based systems or fixed input methods, while hybrid approaches combine multiple techniques. For example, web services like Google’s Dialogflow are used combining machine learning with rule-based methods^54,55^. The dialog management module processes user input and manages the conversation, linking inputs to appropriate responses. Two main data management types are employed: static management, using pattern matching or rule-based systems, and dynamic management, utilizing machine learning or web services for context switching and intent identification^13^. Most chatbots employ one or more of three types of data repositories: the medical knowledge repository (sourced locally or from online platforms like Wikipedia)^18,56^, user data storage for response customization^57,58^, and conversation scripts^17^, which are often used in pattern matching. By integrating chatbots into the management of infectious diseases, health care providers can enhance patient education, improve access to information, and streamline communication, ultimately leading to better health outcomes. Throughout the COVID-19 pandemic, agencies such as the Centers for Disease Control and Prevention (CDC) and the World Health Organization (WHO) have adopted chatbots to distribute information, promote safe practices, and deliver emotional support. For example, the CDC developed “Clara” using machine learning and natural language processing^14^. Clara, known as the “coronavirus self-checker”, was launched to assist individuals in determining the appropriate actions if they experience potential COVID-19 symptoms. This chatbot was created in collaboration with the CDC Foundation and Microsoft’s Azure Healthcare Bot service^14,59^.

## Ethics, data privacy, and algorithmic bias

Although the application of AI in healthcare holds great promise for improvement, it also presents significant ethical challenges, such as (1) obtaining informed consent for data use, (2) ensuring safety and transparency, (3) protecting data privacy (4) addressing algorithmic fairness and biases^60^. Indeed, as AI becomes integral to personalized medicine, protecting patient data and ensuring fairness are critical. The vast collection of sensitive health information requires stringent data privacy measures, such as encryption and controlled access, to prevent unauthorized use^61^. Authorities are working to create clear guidelines for the safe, ethical deployment of AI, with ongoing collaboration among healthcare professionals, developers, and policymakers essential to navigating AI’s evolving role in medicine. Regulatory bodies like the FDA in the US and the Medicines and Healthcare Products Regulatory Agency (MHRA) in the UK are responsible for monitoring AI use in healthcare, ensuring compliance with safety, privacy, and quality standards^62^. They conduct audits and inspections to ensure that stakeholders follow necessary guidelines. The General Data Protection Regulation (GDPR) sets stringent obligations for data privacy, while the European Data Act, effective from January 2024, regulates fair access and use of data, including health data. At the same time, the WHO emphasizes principles like transparency, safety, and equity, guiding states in creating regulations to ensure ethical AI deployment in healthcare and promoting oversight and accountability mechanisms^62^.

Additionally, AI models must be developed with diverse, representative data to avoid perpetuating biases in healthcare. Fairness in healthcare is about providing equitable access to care, resources, and outcomes for all patients, regardless of their background or social status. In AI-driven healthcare, achieving fairness requires developing algorithms that are free from bias and capable of delivering accurate diagnoses and treatments to all patient groups^63^. Biases can arise from data used to train AI, the design of algorithms, and the ways in which healthcare professionals and patients interact with AI systems. To address these challenges, it is essential to use diverse and representative data, conduct regular audits, and provide education to clinicians and patients on potential biases and how to navigate them^63^.

## AI for drug discovery

**Fig. 3:**
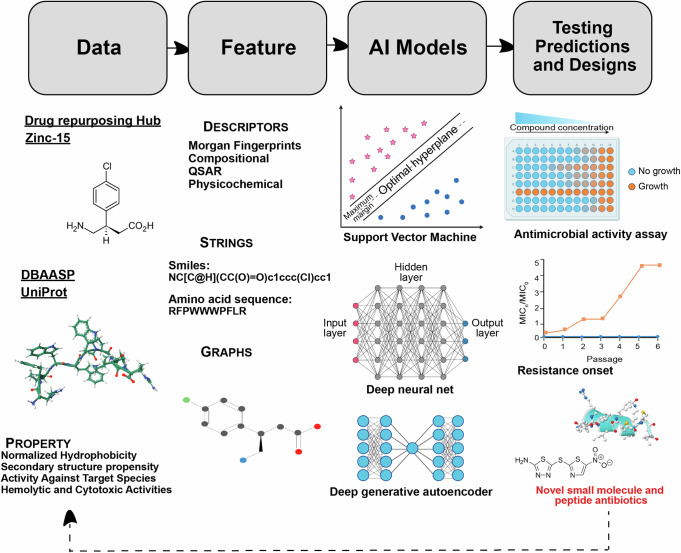
Using AI to find new antibiotics. Various structural and property descriptions of small molecules and peptides from available databases are used to train predictive and generative AI models. These models can be trained on different input representations. AI predictions and designs are then tested in wet lab for antimicrobial activity and resistance onset against different pathogens. This workflow can be iteratively executed, where the experimental results from each iteration can inform the training and testing of the AI models in subsequent iterations.

Beside their application in diagnostics and infectious diseases treatment, AI-based methods have revolutionized our ability to predict biomolecular properties and structures, while also enabling the generation of new active compounds (Fig. 3)^11,21,22,64^. Moreover, ML-driven modeling offers a pathway to bypass certain limitations associated with traditional drug discovery methods, facilitating the rapid design and application of antimicrobial molecules^15,19,23,65–68^. With the increasing diffusion of antimicrobial resistant species and the inherent challenges in developing new drugs, both scenarios that greatly contribute to the failure of antibiotic therapies, there is a pressing need to expedite the design of novel antimicrobials^2,10,19–22,69^.

Antimicrobial peptides (AMPs) and small molecules both serve as therapeutic agents for treating bacterial infections. However, their identification and translation into clinical use has been hindered by the time constraints of traditional high-throughput experimental screenings, as well as challenges such as toxicity, poor stability, limited cellular penetration, and synthesis cost^70,71^. Both AMPs and small molecule drugs have hurdles to clinical approval. However, the science behind the rational design of small molecules is more mature and thus they constitute the vast majority of approved antibiotics. Natural AMPs are increasingly being considered as promising substitutes for conventional antibiotics and as potential successors in antimicrobial therapy, owing to their diverse structures and functions, potent antibacterial properties, and minimal propensity to induce resistance. Thus, synthetic designed AMPs also hold great promise as future therapeutics^72,73^, and examples of AMP-based antibiotics such as polymyxin B and bacitracin are also in common clinical use^74,75^.

Machine learning methods have been employed for decades for the purpose of biological activity prediction^23,76–80^. These techniques are broadly referred to as quantitative structure-activity relationship (QSAR) models. The underlying machine learning model used may vary [e.g., support vector machine (SVM), naïve Bayes, neural network, etc.], but the framework involves learning the relationship between certain input features (usually molecular or peptide descriptors) and the activity potency (or, more generally, any chemical property) (Fig. 3). For fighting AMR and identifying new potential drugs, predictive ML models have been shown to be effective in predicting biological activity of both small molecule antibiotics^5,20,81^ and AMPs^22,79,82^. They may also be used to predict secondary properties relevant to drug candidates such as solubility, toxicity, etc.^83^.

More recently, deep learning models which rely on automatically learned features instead of pre-defined molecular descriptors have been developed for predicting various properties of small molecules and peptides. Typically, these encode a string representation (e.g., SMILES, SELFIES^84^, amino acid sequences) of a molecule or peptide into a latent embedding space of reduced dimensionality which can then be used to train a predictor. Graph neural networks (GNNs) are also a popular choice for molecular property prediction models^85^. These represent the atoms and bonds of a molecule as a graph and can infer global properties from the hidden states of the nodes (and optionally from edges). GNNs may additionally consider modeling three-dimensional features of the molecules. In addition to strings and graphs, DL models can also consider other forms of input representations including pre-defined descriptors, images, natural language text descriptions, and spectrograms (Fig. 3). The architecture of these models can range from convolutional nets, recurrent nets (e.g., long short-term memory networks^86^), message passing neural nets as well as attention-based methods such as Transformer encoders^86^. Prediction models have even been combined with a graph explainablity method to identify the substructures responsible for certain traits, giving insight into otherwise opaque DL methods (Fig. 3)^21,87^.

When tasked with finding drugs with antimicrobial properties, these predictive models can be used to great effect in in silico virtual screening of very large databases due to their speed of inference, compared to that of wet-lab screening or physics-based scoring. For example, this makes it possible to search through vast genomic and proteomic data for possible AMPs^73,86^. It is even feasible to screen every one of the hundreds of billions of possible 6-9 amino acid peptides^77^. AMPs can be sourced from nature or extinct organisms, or synthesized in the laboratory through recombinant or chemical processes. Over 2,603 peptide antibiotics have been computationally identified within the human proteome^73,78,88^, while an additional 323 peptide antibiotics were found encoded in small open reading frames within human gut metagenomes^89^. An extensive computational analysis of the global microbiome, encompassing 63,410 metagenomes and 87,920 microbial genomes, led to the discovery of nearly one million new antibiotic molecules within microbial dark matter, several of which demonstrated efficacy in preclinical mouse models. This recent study represents the largest exploration of biological data for antibiotic discovery to date^78^. Starting from the hypothesis that such antibiotic peptides might be present not only in the human proteome but across the entire tree of life, Maasch et al. applied a machine learning model to search for similar molecules in our closest relatives, Neanderthals and Denisovans, effectively launching the field of molecular de-extinction^22,90^. Building on this work, the team developed a new deep learning model called APEX to mine all known extinct organisms for antibiotics^22^. This approach led to the discovery of antibiotics throughout evolutionary history, including preclinical candidates from the woolly mammoth and other organisms. Notably, over 37,000 sequences were discovered encrypted in extinct organisms. Several of these encrypted peptides worked synergistically at nanomolar concentrations to target pathogens and demonstrated potent anti-infective activity in preclinical mouse models^22,90^.

Also worth mentioning are similar efforts for identifying small molecule antibiotics, Stokes, et al., employed in silico screening on existing and potential drug molecule libraries and successfully reported antibiotic activity of repurposed drug molecules with one demonstrating broad-spectrum activity. The authors trained a message-passing neural network (MPNN) on 2335 molecules to predict binary *E. coli* inhibition (ROC 0.896) and applied it to known molecules meant for either repurposing or hit-identification. MPNNs are a type of GNN which learn by updating local states with information from neighboring nodes and aggregating these to provide a global representation. The molecules with the best predicted activity were then tested in vitro with 51 out of 99 showing inhibition. Subsequently, Liu et al. trained a similar model for screening *A. baumannii* antibiotic candidates using a dataset of 7684 experimentally tested molecules (ROC 0.792)^4,5,91^. They also applied this model to a set of drug repurposing candidates and reported 40 out of 240 empirically validated molecules showed >80% growth inhibition. In summary, this technique can reduce screening time compared to traditional high-throughput screening and is especially useful for drug repurposing.

In order to discover new substances, generative deep learning models have been increasingly employed in the past years. Generative models rely on modeling the distribution of the training data and then sampling new molecules from that learned distribution. Those can be trained using labeled data, unlabeled data, or a combination of both. If the model employs unsupervised or self-supervised learning which does not require (e.g., activity) labels, the models may be trained on large databases of drug-like molecules (e.g., ZINC-15^92^) or bioactive peptides (e.g., UniProt^93^) and therefore have demonstrated robust and improved performance in terms of diversity and novelty of their generations^94^. Like predictive models, these can use different input representations such as graphs or strings. Popular generative architectures are autoregressive decoders, encoder-decoder architectures and generative adversarial networks. The generation of the final molecule can involve building a structure by combining known fragments^95^ or directly generating the output in a recurrent or autoregressive fashion (e.g. using VAEs^69,96^, GNNs^97^, or Transformers^98–100^).

By themselves, unconditional generative models will only reproduce the training data distribution which is not guaranteed to match the target distribution defined by a set of user-selected criteria (activity, toxicity, etc.). One simple method used by Capecchi et al. to generate non-hemolytic AMPs is to fine-tune the broadly-trained model via transfer learning on the target distribution. There, the authors trained an RNN on the entire DBAASP before fine-tuning two models, one on just non-hemolytic peptides active against *P. aeruginosa/A. baumannii* and another on those active against *S. aureus*^79^. Ultimately, 28 peptides were synthesized and tested and eight were active and non-hemolytic^79^. Alternatively, additional feedback or reward signal from a (set of) predictive model(s) is often used to guide generation of the generative model. Many algorithms and methods exist for conditional or controlled generation of artefacts with specific properties. Here, the choices are extensive. Monte Carlo sampling^20,69,101^and conditional VAEs and generative adversarial networks (GANs)^102^ are common approaches. For example, Das, et al. trained a regularized variational autoencoder in an unsupervised fashion on both bioactive and unlabeled peptide sequences and then performed a property-conditional sampling on the learned latent space with additional toxicity filtering from an LSTM model^69^. VAEs encode inputs into a compressed probabilistic latent space which can then be used to generate new data similar to the training by sampling and decoding. Conditional sampling allows the model to draw from a specific sub-distribution within the larger latent space. This approach, without utilizing any existing AMP prototype as a seed for generation, successfully found 7 out of 20 AI-designed peptides to exhibit low to high antimicrobial activity. Of particular importance, two of the seven validated AI-designed AMPs did show broad-spectrum efficacy and low in vitro and in vivo toxicity. Furthermore, those two AI-discovered peptides show high efficacy against *K. pneumoniae* resistant to polymyxin B, an antibiotic of last resort. They also mitigate drug-resistance onset in *E. coli*. The generative DL based approach in that study yielded a 10% success rate and a rapid turnaround of 48 days in discovering novel AMPs with desired properties. Similarly, Swanson, et al., recently employed generative AI that uses Monte-Carlo tree search to design molecules active against *Acinetobacter baumannii*^20^. They identified six new compounds that inhibit the growth of *A. baumannii* and various other phylogenetically diverse bacterial pathogens, out of 58 synthesized and tested designs. Notably, their model also prioritized synthesizability of the generated molecules by building them step-by-step using known reactions and reagents.

In addition to de novo generation, deep generative models also can be employed for molecular optimization (MO) tasks. In that case, the learning method aims to alter an existing structure by reinforcement learning (RL)^103,104^, evolutionary algorithms^3,105^, guiding the search in a learned latent space^106^, or by directly translating to an analog with higher desirability^106,107^. These models may be used for lead generation from hits and can consider additional relevant properties such as toxicity, solubility, bioavailablity, and synthesizability.

When moving to these later stages of drug discovery, traditional computational chemistry methods such as molecular dynamics (MD) are still useful for understanding the mechanisms of binding with targets. MD, and variants such as Markov state models and adaptive sampling, can offer insight into AI model choices or serve to screen/rank candidates^69,108^. Conversely, DL can also be used to analyze simulated dynamics as in Zhu et al. in which a convolutional VAE and CNN were used to cluster and compare data from MD^109^.

Several challenges remain in applying AI to drug discovery. While unlabeled chemical data is abundant, high quality labeled data is still often siloed in proprietary databases or too difficult or expensive to collect. Multimodal models, which integrate multiple data modalities at once to augment understanding, show promise for creating even better models but require more large, quality datasets. Additionally, current models struggle to generate candidates that satisfy a large number of secondary properties at once which is necessary for later stages of drug development. Finally, it is important to understand the outputs of AI models to probe their limits and prevent “hallucinations”.

## Conclusions and future perspectives

As AI technology advances, it holds great promise for disease diagnosis and drug discovery, offering tailored interventions to combat antibiotic inefficacy. AI-based models’ ability to analyze vast clinical and experimental datasets may enable precise disease diagnosis, treatment prognosis, and the prediction or development of novel antimicrobial compounds. This transformative potential extends to critical care settings, where AI models enhance diagnostic accuracy and guide personalized treatment strategies, ultimately improving patient outcomes. On the other hand, ML and DL are speeding up the discovery of new antibiotics, helping to strengthen the preclinical antibiotic pipeline. This is crucial as traditional drug discovery methods have lagged, and the rise of drug-resistant microbes has led to less effective treatments. Indeed, as AI integration expands, its role in refining infection treatment regimens and enhancing antibiotic stewardship is poised to become indispensable in the fight against AMR.

In this context, it is important to also recognize that, despite the advancements of cutting-edge algorithms, limitations in data accessibility still present challenges, impacting their ability to perform effectively across diverse patient populations and clinical scenarios. Particularly, ethical concerns such as informed consent, data privacy, and algorithmic bias must be addressed. AI models should be developed using diverse, representative datasets to avoid perpetuating bias and ensure fair and accurate outcomes for all patient groups. Indeed, AI models for AMR diagnosis, treatment, and new antimicrobial discovery are often trained on imbalanced datasets, which can suffer from low reliability. These models are frequently based on inherently biased datasets, capturing data from only certain patient groups or specific biological assays. Such biases may reflect human cognitive biases and social influences in data curation. AI models trained on these biased datasets may lack the ability to generalize beyond their training domain. As data curation activities grow, AI models will benefit from high-quality data management and sharing protocols. Indeed, when building a dataset, it is essential to consider various parameters, including size^110^. Additionally, while large datasets can provide detailed insights and support advanced modeling, smaller, well-annotated, and accurate datasets may offer more valuable insights by avoiding excessive noise. The focus should be on selecting data that aligns with specific objectives and designing systems to generate, collect, and analyze raw data efficiently, using automated and streamlined workflows. For example, the study of metalloantibiotics is hindered by the lack of a dedicated public database with a consistent representation as they are not accurately represented by SMILES^111,112^. To address this gap, there is a need for dedicated funding and efforts to create robust datasets, which will contribute to unlocking AI’s full potential in combating infectious diseases and drug resistance^110^. Moreover, research on the development and application of AI in AMR is still underway, and the “black-box” nature of deep learning algorithms can raise concerns about their trustworthiness.

Therefore, the accessibility of data for investigators significantly influences both the quantity and quality of research on AI model development, particularly for diagnosis and treatment prediction^113^. When health data is readily available, researchers can build AI models that are more accurate and reliable across a wide range of patient populations. In contrast, limited access to well-rounded and representative datasets restricts the development of robust algorithms, leaving biases in underrepresented groups unaddressed^113^. Providing investigators with access to diverse, high-quality data is essential for developing AI models that perform well across various populations, minimizing algorithmic bias and improving overall outcomes.

Looking ahead, we anticipate the utilization of AI models trained on expansive, superior-quality datasets derived from high-throughput technologies and genomic/expression databases. These models will aid in constructing systematic frameworks for swift disease diagnosis and suggesting novel antibiotics with unique mechanisms of action. For antibiotic discovery, this means going beyond hit generation proofs-of-concept as well as considering more secondary properties besides toxicity and integrating AI in the later stages of drug development. The solution to these multifaceted problems involves multiple AI models working together, infused in all the different stages discussed here. Furthermore, collaborating with healthcare professionals, developers, and regulatory bodies will be essential in ensuring AI is used responsibly, with ongoing efforts to maintain fairness and transparency. Regulatory guidelines are likely to play a critical role in safeguarding patient data and promoting safe AI deployment.
